# Modeling and Statistical Analysis of the Spatio-Temporal Patterns of Seasonal Influenza in Israel

**DOI:** 10.1371/journal.pone.0045107

**Published:** 2012-10-08

**Authors:** Amit Huppert, Oren Barnea, Guy Katriel, Rami Yaari, Uri Roll, Lewi Stone

**Affiliations:** 1 The Gertner Institute, Chaim Sheba Medical Center, Tel Hashomer, Israel; 2 Biomathematics Unit, Department of Zoology, Faculty of Life Sciences, Tel-Aviv University, Tel-Aviv, Israel; 3 The Porter School of Environmental Studies, Tel-Aviv University, Tel-Aviv, Israel; 4 Department of Mathematics, ORT Braude College, Karmiel, Israel; University of Oxford, Viet Nam

## Abstract

**Background:**

Seasonal influenza outbreaks are a serious burden for public health worldwide and cause morbidity to millions of people each year. In the temperate zone influenza is predominantly seasonal, with epidemics occurring every winter, but the severity of the outbreaks vary substantially between years. In this study we used a highly detailed database, which gave us both temporal and spatial information of influenza dynamics in Israel in the years 1998–2009. We use a discrete-time stochastic epidemic SIR model to find estimates and credible confidence intervals of key epidemiological parameters.

**Findings:**

Despite the biological complexity of the disease we found that a simple SIR-type model can be fitted successfully to the seasonal influenza data. This was true at both the national levels and at the scale of single cities.The effective reproductive number *R_e_* varies between the different years both nationally and among Israeli cities. However, we did not find differences in *R_e_* between different Israeli cities within a year. *R*_e_ was positively correlated to the strength of the spatial synchronization in Israel. For those years in which the disease was more “infectious”, then outbreaks in different cities tended to occur with smaller time lags. Our spatial analysis demonstrates that both the timing and the strength of the outbreak within a year are highly synchronized between the Israeli cities. We extend the spatial analysis to demonstrate the existence of high synchrony between Israeli and French influenza outbreaks.

**Conclusions:**

The data analysis combined with mathematical modeling provided a better understanding of the spatio-temporal and synchronization dynamics of influenza in Israel and between Israel and France. Altogether, we show that despite major differences in demography and weather conditions intra-annual influenza epidemics are tightly synchronized in both their timing and magnitude, while they may vary greatly between years. The predominance of a similar main strain of influenza, combined with population mixing serve to enhance local and global influenza synchronization within an influenza season.

## Introduction

Seasonal influenza outbreaks are a serious burden for public health worldwide. Seasonal influenza is mainly a self-limiting disease, and in most patients results in only moderate illness without need for medical treatment. Nevertheless, it is estimated to cause morbidity to millions of people each year. In addition, influenza poses a major risk to chronic patients of all ages especially the elderly, to whom it causes more severe morbidity and is associated with a higher death rate [1]–[3]. The global mortality from the disease is estimated at 250,000 to 500,000 cases annually [4], [5]. Furthermore, the economic burden of seasonal influenza is estimated to be 11 billion US dollars a year in the US alone [6]. This includes morbidity, mortality, hospitalizations and absenteeism from work and school. As early as 1952 the WHO established the Global Influenza Surveillance Network. However there are major problems regarding the reliability of influenza data [7], [8]. The major difficulty is that the disease has no clear-cut clinical signs and can be easily confused with other respiratory illnesses having similar symptoms [9]. In addition, influenza illness is often slight or moderate and a significant number of infected individuals are asymptomatic, so that many individuals with the disease do not seek any medical care. In order to better estimate the burden of influenza, different sources of influenza data have been used to study the disease, such as Influenza-Like Illness (ILI) diagnoses, virus isolations, death records from pneumonia and influenza, physician visits and web search queries [2], [4], [10]–[12].

**Figure 1:**
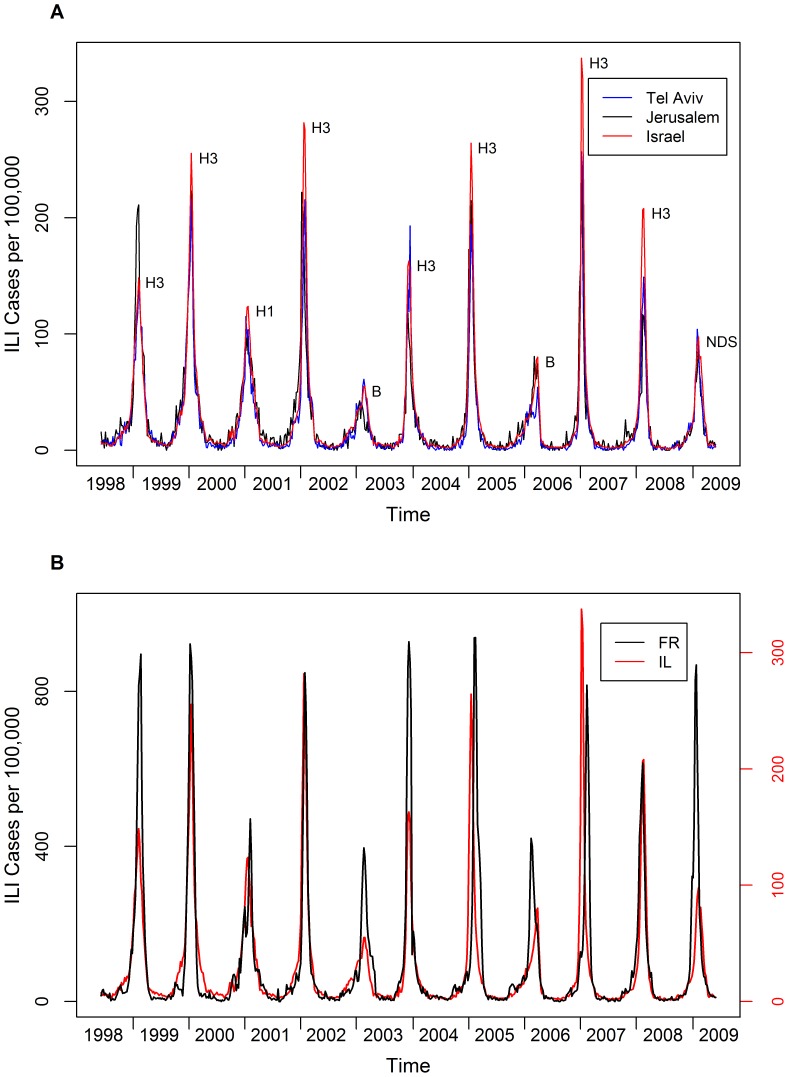
ILI incidence in Israel and France, 1998–2009. (A) Weekly number of ILI cases per 100,000 Maccabi Health Services members in Israel, Tel Aviv and Jerusalem. The text next to the peaks indicates dominant subtype (NDS = no dominant subtype). (B) Weekly number of ILI cases per 100,000 people in Israel (Maccabi Health Services members) and France (sentinel clinics patients). Note different scales for each time series, as French incidence is ∼3 times higher than Israeli incidence.

Influenza is predominantly seasonal, with epidemics occurring every winter, but the severity of the epidemic and the exact timing of the outbreak vary substantially between years (see figure 1). Influenza has also been studied with respect to its geographic spread at different scales. Viboud et al. (2006) have studied the spread of influenza across the United States and found that there is higher synchrony between more populous states [12]. In other studies Viboud et al. (2004) and Chowell et al. (2008) analyzed the synchrony on a global scale comparing outbreaks in the US, France and Australia [13], [14] and found high synchrony between the US and France and no synchrony between the Northern and Southern hemispheres (even after adjusting for the two hemispheres being out of phase). In the smaller spatial scale of France, Bonabeau et al. (1998) demonstrated how the disease rapidly spreads across the country, and concluded that when modeling the initial spread of influenza it is sufficient to assume global homogeneous mixing to capture the dynamics. Geographic heterogeneities and density dependence affect the dynamics of the disease around local outbreak peaks [15].

Israel provides a unique opportunity to study the spatial spread of influenza at a small scale since the country is only approximately 22,000 km^2^. We use a highly-detailed influenza database, both temporally and spatially (see data section) collected in Israel for over 11 years. The data composed of ILI diagnoses in a subset of some 23.8% of the Israeli population. We initially examine the ILI data on a national level (i.e. aggregated data) to understand the year to year variability of influenza epidemics both in their timing and magnitude. In the second part of the paper we examine spatial aspects of influenza in Israel with emphasis on the spatial synchrony. The following patterns are of particular interest: i) Differences in ILI dynamics and key epidemiological parameters between the major cities in Israel (in time and space) ii) Synchrony between different Israeli cities within a year iii) Comparing the “local” Israeli synchrony with the intercontinental synchrony of influenza outbreaks tested on the time series of Israel and France.

### Modeling Approach

**Figure 2:**
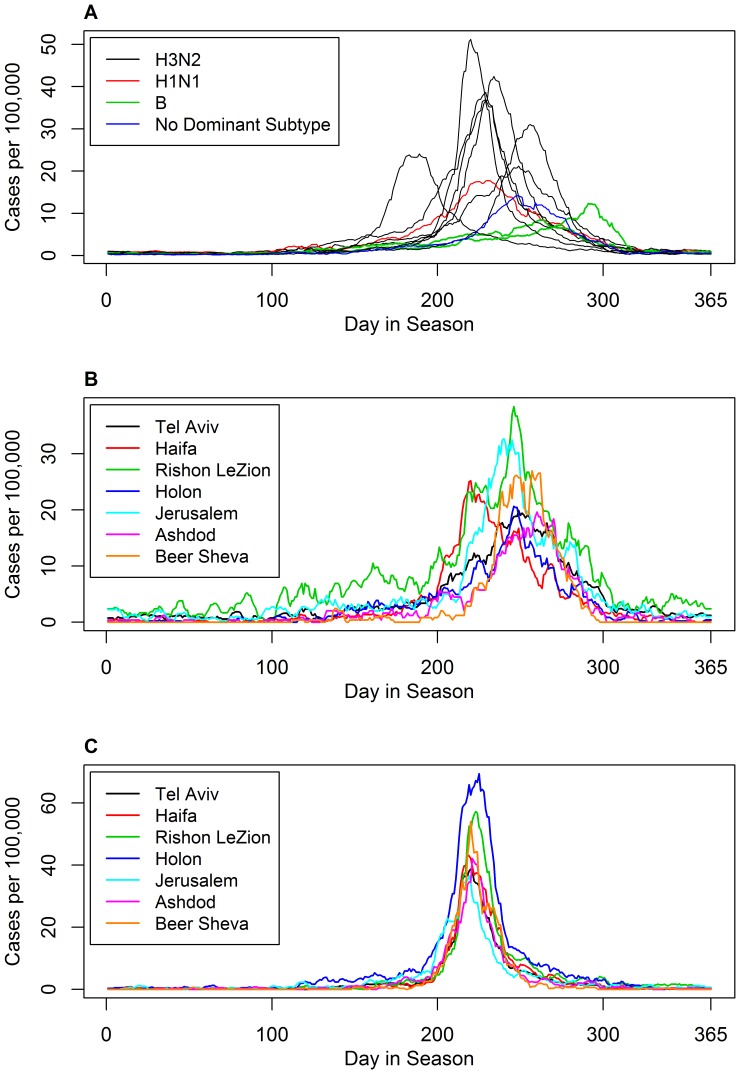
Synchronization of ILI incidence between seasons and within seasons. Top (A): superimposition of eleven seasons of daily ILI incidence in Israel. Starting date is June 1^st^. Middle (B): superimposition of ILI incidence in seven cities in the 1998–1999 season where *R_e_* = 1.2 was low and the correlation between the different cities is relatively weak. Bottom(C): ILI incidence in the same seven cities during the 2006–2007 season where *R_e_* = 1.6 was high and the correlation between the different cities was strong.

Figure 1 displays the time series of influenza outbreaks (ILI cases) over eleven years when aggregated spatially over all of Israel. There is considerable variation between years in both the peak height of the outbreak, the total attack rate, and the times at which the epidemics reach their maxima during the winter months (December to March (figure 2A). The high quality of the entire dataset both in terms of temporal and spatial resolution (see data section), is a key feature that motivates the following modeling analysis. The data were analyzed and modeled both: i) at the national level, using the total number of ILI cases aggregated over the entire country (figure 1), and ii) in the seven largest cities of the database (in terms of population size). These cities provide a picture of the spatial variation of influenza across the country.

A discrete time age-of-infection SIR epidemic model, as formulated in Katriel et al, (2011) [16], is used to fit the Israeli ILI data and estimate epidemiological parameters in order to gain a better understanding of the influenza dynamics (see Methods for model description). The SIR framework assumes that individuals within a population can be divided into three categories or compartments: Susceptibles, Infected or Recovered. Disease transmission within the population is modeled by tracking the changes in numbers of individuals within these compartments. Although SIR-type models have become almost the gold standard for modeling bacterial and viral respiratory infectious diseases, it is to some degree an oversimplified model for influenza due to the virus's ability to rapidly mutate giving rise to its characteristic antigenic drift [17], [18]. Earn et al (2002) argue that “it seems impossible to avoid a much greater degree of model complexity [than the SIR]. The primary obstacle to simple compartmental modeling of flu is antigenic drift.” [7] In order to bypass the difficulties of modeling continuous evolutionary changes in influenza we have chosen to model individual years separately as single influenza outbreaks, a practice that may be found in the important studies of Baroyan et al (1971) [19] in the USSR, Spicer (1979) [20] in the UK and recently by Chowell et al (2008; 2010) for the US, France, Australia and Brazil [14], , Cintron-Arias et al (2009) who modeled US epidemics [22] and van Noort et al (2011) who used the same approach to modeling influenza time series as single consecutive outbreaks in the Netherlands, Belgium and Portugal [23]. Our analysis goes further than these studies in the manner that it uses a specially formulated statistical approach for estimating key epidemiological parameters.

The basic reproductive number *R*_0_ is an important and widely employed index for quantifying individual epidemic growth rates [24]. *R*_0_ is defined as the average number of people infected by a typical individual over the disease infectivity period in a totally susceptible population. In general, it is extremely difficult to estimate *R*_0_ because the initial population is rarely totally susceptible. Most studies of influenza therefore only attempt to estimate the “effective *R*_0_”, or *R_e_*, which is a composite index: *R_e_ = R*_0_ • *S*_0_, where *S*_0_ is the proportion of the population who are susceptible at the beginning of an outbreak. The effective reproduction number *R_e_* should be interpreted as the average number of people an infected person infects during the course of their illness in a population, a fraction *S*_0_ (*S*_0_<1) of which is susceptible. But *R_e_* tells us little about *R*_0_ since *S*_0_ (an important variable in its own right), is difficult to estimate directly [25]. There are many methods documented in the literature for estimating *R_e_* and nearly all of them calculate the rate of exponential growth of the infected population in the first phase of an outbreak [26]–[28]. Recently there have been several methods developed for estimating both *R*_0_ and *S*_0_ which are usually more complex and in most cases require fitting an SIR type model to the data [22], [23], [29]–[31]. Certainly there would be a major advantage in having the ability to separate out these two parameters because they have very different biological meanings. In the approach used here, we take advantage of the fact that for the Israeli dataset, the entire epidemic curve is available for analysis and not just the initial phase. We are thus able to fit the age-of-infection SIR model to the full epidemic curve to obtain estimates of both *R_0_* and *S_0_* for each epidemic outbreak. In Table 1 the estimates of *R_e_* obtained using the entire curve (see methods) are given together with estimates of *R_e_* calculated using the classical method [32] which estimate the exponential growth of the number of infected people. These two estimates of *R_e_* are significantly correlated (r = 0.91, p = 0.0007).

**Table 1:** Basic epidemiological and population data for eleven ILI seasons in Israel.

Season	Maccabi Members	Total Cases	Attack Rate	Peak Cases (date)	S_0_ (95% CI)	R_0_ (95% CI)	R_e_ (95% CI)	R_e_ (exp. growth)
(1) '98–'99	1,376,455	22,898	16.6%	292 (Feb. 4)	37.9% (32.3–45.5)	3.18 (2.54–3.84)	1.20 (1.17–1.24)	1.2
(2) '99–'00	1,421,000	31,438	22.1%	525 (Jan. 15)	39.5% (36.1–43.9)	3.22 (2.82–3.63)	1.28 (1.25–1.31)	1.27
(3) '00–'01	1,497,259	23,104	15.4%	266 (Jan. 16)	40.4% (33.9–50.1)	2.90 (2.23–3.56)	1.17 (1.14–1.21)	1.17
(4) '01–'02	1,553,547	31,354	20.2%	660 (Jan. 20)	28.4% (26.6–30.4)	4.85 (4.39–5.32)	1.40 (1.37–1.44)	1.39
(5) '02–'03	1,600,097	13,805	8.6%	135 (Feb. 19)	-	-	-	
(6) '03–'04	1,630,116	21,303	13.1%	392 (Dec. 6)	28% (25.7–30.9)	4.62 (4.08–5.16)	1.30 (1.27–1.33)	1.42
(7) '04–'05	1,666,194	28,549	17.1%	644 (Jan. 15)	24.3% (22.8–26.1)	5.7 (5.14–6.26)	1.40 (1.36–1.44)	1.52
(8) '05–'06	1,697,196	14,818	8.7%	210 (Mar. 18)	-	-	-	
(9) '06–'07	1,728,824	29,952	17.3%	885 (Jan. 6)	19.6% (18.9–20.5)	8.16 (7.62–8.71)	1.62 (1.58–1.66)	1.58
(10) '07–'08	1,767,397	25,801	14.6%	548 (Feb. 11)	23% (21.1–25.6)	5.76 (5.06–6.52)	1.382 (1.35–1.42)	1.36
(11) '08–'09	1,819,376	17,560	9.7%	257 (Feb. 4)	21.5% (18.9–25.2)	5.76 (4.75–6.76)	1.24 (1.20–1.28)	1.22

Values for *S*_0_, *R*_0_ and *R*_e_ are missing for the influenza B seasons (2002–3 and 2005–6) due to poor fit of the SIR model. Attack rates, *R*_0_ values and *S*_0_ values are calculated under the assumption of a reporting rate of 10%.

Our methodology gives estimates of *R_0_* and *S_0_* under the assumption that all influenza cases in the population are reported and thus that the ILI time series are a product of a surveillance system with a perfect, or 100%, reporting rate. However, such a situation is never the case in practice. If we assume that the reporting rate of the surveillance captures the proportion ***r*** (0<*r*<1) of true influenza cases, then we refer to our estimates in the presence of partial reporting as 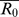 and 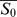 while the desired values under perfect reporting are referred to as 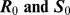. They are related as follows [25]:
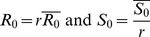
(1)


Since the true reporting rates (*r*) of surveillance systems are rarely quantified accurately, they become an important limiting factor when trying to estimate these key epidemiological parameters. However, for well managed surveillance systems, it can be assumed that ***r*** is reasonably constant over extended times, in which case it would be possible to capture the relative values and trends of these parameters as they change in time. Unfortunately there have been no studies of the reporting rate stability of the Maccabi data. We have compared the data to another independent Israeli surveillance and found almost identical trends over the same period indicating the reporting rate has changed little. Nevertheless, as we note in the Data section, there was a change in the ILI coding in 2002, which might possibly have changed the reporting rate in that year, presumably to a small degree.

In our data, it was found that on average 1.5% of all Maccabi members were infected with influenza annually (see table 1 for full list of attack rates). However, in the US, average overall attack rates are estimated to be 10–20% [33], [34], and France some 12–15% [35]. This, together with discussions with the Israeli Ministry of Health, motivated our setting of the reporting rate to 10% (r = 0.1), yielding an attack rate in Israel (adjusted to 15%) consistent with that reported in the literature for other countries [8], [14]. In this case, the average estimates of the true *R_0_* in Israel using equation 1 should be *R_0_*∼4.9 which is close to the rough calculation of Katriel and Stone (2010) who estimated *R_0_*∼3.75 [25].

We note that the parameter *R_e_* has the interesting property that it is entirely independent of the reporting rate since:
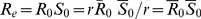
(2)


Thus estimations of *R*_e_ remain unaffected by spatial differences or temporal changes in reporting rates.

## Results

### Temporal Features

**Figure 3:**
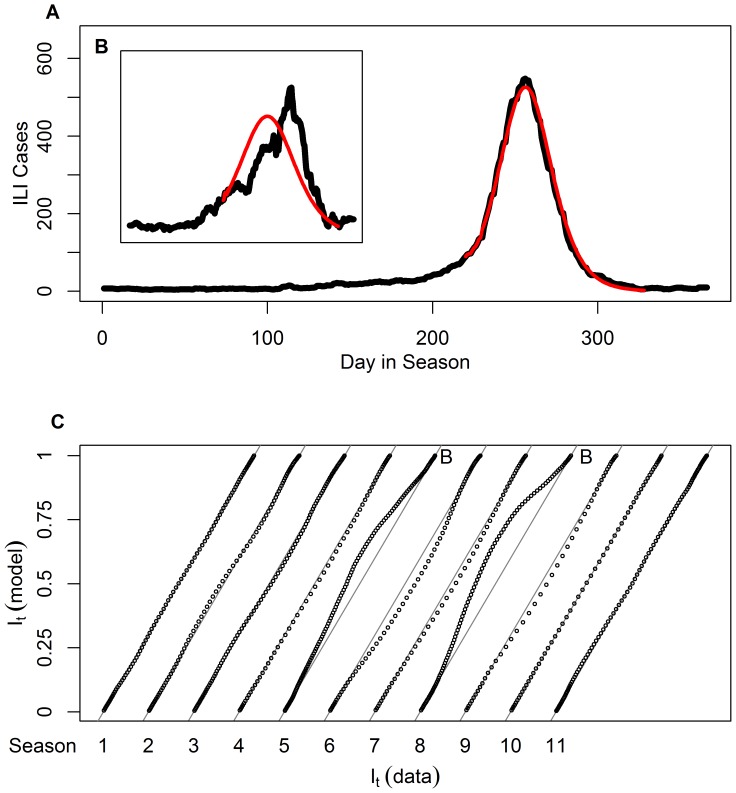
Fits of SIR models to observed ILI incidence. Top (A): model fits to the 2007–2008 season (main) and the 2002–2003 season (inset (B)). Bottom(C): model fits to all eleven seasons 1998–1999 to 2008–2009 where observed cumulative infectives is plotted as a function of model SIR cumulative infectives (dots). Diagonal lines indicate a perfect fit of the model to the data; Day 1 in each season corresponds to June 1^st^. Time length of each epidemic varies from season to season due to differences in the start and end date of the best-fitting model.

The time course of each of the outbreaks in the aggregated Israeli time series was fitted using the discrete-time SIR model (see Methods). In general the model accurately reproduced the time course of the Influenza A epidemics as demonstrated by the simulation run shown in figure 3A based on data for the year 2007–8. Figure 3C provides a more general picture by displaying model fits for each epidemic in all eleven years as compared to the observed data, based on a cumulative plot, similar to a Q-Q plot (cf [30] and methods). The cumulative incidences of the observed data are plotted on the x-axis, while the cumulative incidence produced by the SIR model is plotted on the y axis. The solid straight diagonal lines are reference lines which connect all points (*I*_t_, *I*_t_) of the observed data. The lower points in each series represent the early stage of the epidemic and the top points represent the late stage of the epidemic. Perfect fits between model and data would result in all points falling on the diagonal reference lines. This is to some degree achieved in the fits of the Israeli Influenza A data. The fits of the Influenza B seasons, however, as seen also in figure 3B, are of a lower quality.

**Figure 4:**
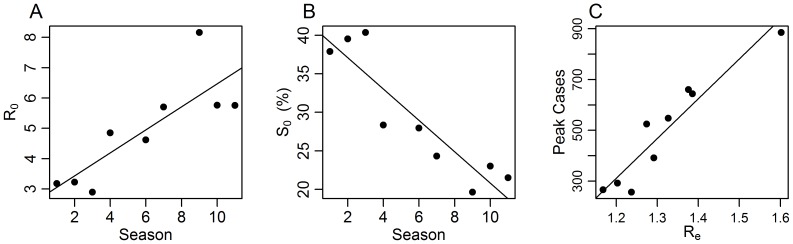
Correlations of several epidemiological parameters in the ILI dataset. The relationship between season (time) and *R*_0_ (A), season and *S*_0_ (B), *R*_e_ and maximum number of ILI cases at the peak of the outbreak (C). In these analyses we used only A seasons (n = 9).

Also included in figure 1 is a plot of the well-studied influenza (ILI) dataset collected in France (see figure 1B and data section for details) which will be used for the purposes of a comparative study with Israel. We found that the French epidemic data (figure 1B) could also be modeled with good accuracy by the SIR fitting procedure. However, for both the Israeli and French datasets, we were unable to fit influenza B years with the same level of accuracy, since the epidemic curves corresponding to influenza B outbreaks were more asymmetric than the standard SIR model could accommodate for (figure 3B). Intriguingly, when correlated against its year the estimates for 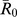 in the Israeli aggregated data showed a significant (p = 0.006, *R* ^2^ = 0.69) long-term increase over the eleven years (figure 4A).

The estimates for *R*_0_ (based on r = 0.1) varied between the lowest *R*_0_ = 2.95 in 2000–2001 and a maximum of *R*_0_ = 8.16 during the 2006–2007 outbreak, with an average of *R*_0_ = 4.9 (see table 1 for full details). We note that it is unlikely that the increase in *R_0_* is due to an increase in reporting rates over this period. Had there been an increase in reporting rate, one would expect a corresponding increase in attack rate over the time-period, but this does not appear to occur. In addition the analysis was repeated after excluding the first years (where the coding system was different) and the trend remained (see figure 4). Interestingly, Spicer (1979) [20] also noted an increase in transmissibility (equivalent to an increase in *R_0_*) with time after a new strain of H2N2 appeared: “transmissibility was low in the early stages of the introduction of the new Asian (H2N2) virus subtype in 1957 and the Hong Kong (H3N2) virus subtype in 1969–1970 and high for some years after. This is biologically plausible if the virus is adapting to new conditions of spread.”

While *R_0_* exhibited a long-term increase over the years, the fraction of susceptibles *S*_0_ showed a significant decrease with time (p = 0.0006, r = −0.91) as shown in Figure 4B. The maximum fraction of susceptible was found to be *S*_0_ = 40.4% during 2000–2001 while the minimum was *S*_0_ = 19.6% obtained in 2006–2007; the average being *S*_0_ = 29.2%.

The average *R*_e_ for influenza A in Israel was found to be *R*_e_ = 1.32, was lowest with *R*_e_ = 1.17 in 2000–2001 (characterized by a dominant H1N1 virus), and highest with *R*_e_ = 1.6 in 2006–2007. The estimates of *R*_e_ for Israel are very similar to estimates for seasonal influenza in other parts of the world Australia, US and France (the average in all 3 countries was *R_e_* = 1.3) [8], [14], but higher then recent estimates of Chowell et al 2010 for Brazil, USA and France *R*_e_ = 1.06, *R*_e_ = 1.14 and *R*_e_ = 1.14 respectively [21]. While using our model to estimate *R_e_* in France during the same time period gave an average estimate of *R*_e_ = 1.36.

Over the eleven years of this study, both *R*_0_ and *S*_0_ vary considerably (e.g., *R*_0_ varied between 2.95–8.16 while *S*_0_ varied between 19.6–40.4%).It is puzzling why the observed decrease in the susceptible fraction of the population over time is balanced out by the increase in the reproductive number *R*_0_ so that the product *R_e_ = S_0_•R_0_* changes to a relatively limited degree and is always slightly larger than unity (figure 4 A, B). The statistical analysis also showed a significant and high correlation r = 0.95 (p≪0.0001, N = 9, all influenza A seasons), between the magnitude of *R*_e_ and the number of ILI cases in the smoothed peak of the epidemics (see figure 4C) in the aggregated national data.

### Spatial aspects of influenza in Israel

One of the most striking features regarding the dynamics of influenza in Israel is the strong spatial synchronization. Figures 1 and 2, show very clearly that the time series of major cities in the country are highly correlated. In order to quantify how the ILI varies spatially across the country, we focus on the two main aspects of the ILI data: the magnitude of the outbreaks in the different cities and the timing or temporal differences in the occurrence of the outbreaks which also varies spatially.

### The variation of R_e_ across Israel

For the purposes of examining spatial differences in the magnitude of influenza outbreaks across Israel we make use of *R_e_* as an index for epidemic intensity [14]. Given that *R*_e_ is a measure that is independent of reporting rate, it is useful for conducting comparisons especially since there are indications that the reporting rates of various cities can be quite different (see data section).

An interesting outcome of our analysis is the finding that there are no statistically significant differences in *R_e_* between the different cities **within a year** (ANOVA test, p = 0.46, F = 0.97). In contrast *R_e_* varies (both nationally and among the Israeli cities) between the different years in a manner that is highly statistically significant (ANOVA test, p = 1.65×10^−13^, F = 17.7). Our results are consistent with previous studies, as for example [13] who conclude for the US, France and Australia that “while the average inter-pandemic *R_p_* seems rather invariant across geographical locations at around 1.3 there is substantial year to year variability around this average”.

### Quantifying the spatial synchrony in Israel

As figure 1 shows, the ILI cases from the two major Israeli cities, Tel Aviv and Jerusalem, appear to be tightly synchronized with a correlation of r = 0.91 (p≪0.001). Both cities are also highly correlated to the spatially aggregated Israeli ILI data having respective correlation coefficients r = 0.98 (p≪0.001) and r = 0.92 (p≪0.001). It is thus not surprising that the attack rates (i.e., the total number of cases per year) of Tel Aviv and Jerusalem are also correlated to the attack rates in Israel with a correlation coefficient of r = 0.92, (p≪0.001) and r = 0.91 (p≪0.001) respectively.

To explore synchrony further we examined whether the timing of the influenza outbreaks observed in Tel Aviv and Jerusalem are more synchronized than expected by chance. Two different tests were devised:

- **Phase Analysis:** The Tel Aviv and Jerusalem time series were superimposed and the peak date of each epidemic in each time series was identified. In the phase analysis [36] we let Δ_i_ represent the time difference between the peak of the outbreak in Tel Aviv and that in Jerusalem for the i'th year, and calculated
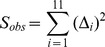
(3)for the observed data. In step two, the annual outbreaks of Jerusalem were randomly reshuffled over the eleven years [37]. The reshuffling required breaking up the Jerusalem time series into eleven separate years (or outbreaks) and then randomly reordering their sequence of occurrence. 
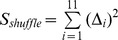
 was then calculated. This was repeated N = 100,000 times to obtain the distribution of *S_shuffle_*. The index *S_shuffle_* was found to be larger than the observed value *S_obs_* in 99,985 of the N = 100,000 reshufflings. Therefore, Tel Aviv and Jerusalem are significantly synchronized (p<0.00014) in terms of the timing of their peaks. An analysis of the nine influenza A seasons (i.e., excluding from the analysis years dominated by influenza B) gave very similar results, with p<0.00024.
- **Correlation Analysis:** Similar to the above test, the correlation *r_obs_* between the Tel Aviv and Jerusalem time series of infectives was measured for both the observed and reshuffled time series. Again, the reshuffling involved breaking up the Jerusalem time series into eleven separate years (or outbreaks) and then randomly reordering their sequence of occurrence. We generated N = 100,000 randomized Jerusalem time series and calculated their correlations *r_shuffle_* with the Tel Aviv time series. This procedure gives the probability distribution of *r_shuffle_*. We found that the correlation between Tel Aviv and the observed Jerusalem time series was higher than the correlation calculated from the randomized Jerusalem time series in all 100,000 cases. This occurred both when all 11 seasons were analyzed and when influenza B seasons were excluded from the analysis. The high correlation observed between Tel Aviv and Jerusalem time series is nonrandom and provides strong support for the notion that the cities are synchronized over and above the background synchrony of the irrepressible annual winter outbreaks.

### Variability of the Spatial Synchrony

We found that different years have different characteristic strengths of spatial synchrony. As a reference frame, figure 2A plots a superimposition of all 11 outbreaks occurring over the 11 seasons in the aggregated national data, and gives an indication of the (relative lack of) temporal synchrony *between* years. This should be compared with figures 2B and C, which are plots of the time series for the seven largest cities for the years 1998–9 (figure 2B) and 2006–7 (figure 2C). The former is an example of a year with relatively low spatial synchrony while the latter is the year with the maximum synchrony among the Israeli cities. Comparing the figures it is easy to see that the synchrony within a year (figure 2B and C) is far stronger than the synchrony between years (figure 2A).

### Epidemic Synchronization between Israel and France

To gain further insights into the synchrony dynamics of influenza in Israel, we studied its relationship to a distant European country - France. Figure 1B displays the aggregated ILI time-series of both countries. Visually one observes in figure 1B that the level of synchrony between France and Israel is surprisingly high (with the small exceptions of the 2004–2005 season and the 2006–2007 season where the outbreak in Israel occurs a few weeks before France). The correlation coefficient between the time series was correspondingly high with r = 0.71. The synchrony was enhanced through the appearance of influenza B which was the dominant virus (i.e., the small peaks in 2002–3 and 2005–6) occurring simultaneously in the same years in both countries (see also [38]).

In order to quantify the temporal synchrony between the two countries we again performed the phase and correlation analyses for the timing of the outbreaks. Both tests found Israel and France to be significantly synchronized, in the timing of influenza outbreaks (p = 0.014 and 0.008, phase and correlation tests respectively). Results were still significant when B seasons were omitted (p = 0.037, p = 0.045 phase and correlation tests respectively).

In addition we tested whether the intensity in the outbreaks between the two countries was correlated. The values of both peak heights and of *R*_e_ were calculated and correlated for all the 9 outbreaks between1999–2009. A significant correlation between Israel and France was found in both peak heights (r = 0.6; p = 0.05) and between the values of *R*_e_ (r = 0.75; p = 0.02). The statistical tests indicate that for both Israel and France i) there are very minor differences in the timing of the epidemics (phase and correlation analyses) and ii) large/small outbreaks tend to occur in the same years in both countries. Nevertheless, and as expected, the correlation between the Israeli cities is higher than the correlation between the two countries.

## Discussion

The high quality of the Israeli ILI data has enabled us to explore the spatio-temporal dynamics of influenza in Israel over eleven years. The fact that the simple SIR model can be fitted successfully to data of seasonal influenza A in both the national (aggregated data) level as well as in the scale of single large cities is notable in light of the complex epidemiology of influenza [7]. This is consistent with previous work from the Soviet Union, United Kingdom, France, United States Australia, the Netherlands, Belgium, Portugal and Brazil [14], [20]–[23], [39], [40]. One of the main advantages of using the modeling approach proposed here is the ability to estimate separately the two components of *R_e_* namely *R_0_* and *S_0_*, which limited many previous studies of influenza [24], [34]. We found that over the study period the value of *R_0_* increased in time (figure 4A). Interestingly this increase was “balanced” by a decrease in *S_0_*. One possible speculation that can explain the observed increase in *R_0_* could be the evolutionary adaption of the virus to become a more efficient infector [41]. For example, the new strain of pandemic influenza, the H1N1 swine flu virus, had relatively low transmission during the swine flu pandemic [16], [42]–. However pandemic influenza is potentially far more dangerous because the immunity of the population to the pandemic virus is “expected to be” lower than the circulating seasonal influenza strains (i.e., a high *S_0_*). It is believed that in the future, the H1N1 virus (which is now considered a seasonal strain) will adapt to become a better infector as has happened with previous pandemic and seasonal strains [45]. As mentioned above, in parallel to the increase in *R_0_* the population susceptibility is expected to decrease due to an increase in the population immunity as more of the population is exposed to the new virus strain. The exact mechanisms which lead to the observed negative correlation in *R_0_* and *S_0_* found here needs to be further studied in order to better understand the dynamics of influenza. We note again that the estimate of *R_e_ = S_0_•R_0_* remains independent of reporting rate. Thus even though there is strong under-reporting in our data, the **estimations of** ***R*** **_e_ remain unaffected.** In Israel the value of *R_e_* is strongly correlated to the magnitude of the peak height of influenza outbreaks (figure 4C). Knowledge of *R*_e_ is essential for understanding and controlling the spread of an infectious disease [24], [45]. For instance the proportion of the population which needs to be vaccinated in order to reach herd-immunity is a function of *R_e_*. Recent studies have shown that a reliable estimation of *R_e_* for seasonal influenza can be obtained within a period of 4 weeks after the initiation of the disease [14]. Therefore, it is possible to estimate *R_e_*, in the first few weeks of the season and use this information to predict the upcoming epidemic.

*R*_e_ was also positively correlated to the strength of the spatial synchronization in Israel. We found that in cases where the disease is more “infectious” (i.e., higher *R*_e_) then the outbreaks in different cities tend to occur with smaller time lags (see figure 2B, C). It may be hypothesized that the higher *R*_e_ implies a more forceful infectivity which increases the synchronization and reduces the variability in the timing and magnitude of the peaks between the different cities (see figure 2). It would be an interesting future direction of study to examine the capacity of this hypothesis to explain the observed correlation between *R*_e_ and synchronization by studying simulations of explicit spatial models.

Modeling studies have shown the sensitivity of the severity of influenza outbreaks to small demographic and environmental changes [46], which implies that small difference in environmental factors can cause large differences in the size of outbreaks between different locations. Nevertheless, we see notable resemblance between the time series within a year across large geographical distances (e.g., Israel and France).

Using laboratory-confirmed influenza surveillance data, Finkelman et al (2007) reported large scale co-occurrence of influenza type A and B, and interhemispheric synchrony (i.e., the dominant strain within a season is the same for most of the hemisphere) [38]. Another example of high synchrony between Israel and France can be seen in Figure 1B, where in the outbreak of 2003–4 there is an early epidemic in both countries. Interestingly the 2003–4 season was dominated by a new influenza strain (A/Fujian) which peaked early in many different countries (probably due to the fact that the population immunity to the new strain was low and therefore *S*_0_ was high, leading to an early outbreak) in the northern hemisphere [50]. Another factor leading to the high spatio-temporal synchrony within Israel is due to short travel distances between cities in a small country. Interestingly, spatio-temporal synchrony is high even between Israel and France, despite the much larger geographic scale. It is possible that here to, a single dominant influenza strain prevails in both countries in each season and air-travel between the countries aids temporal synchrony.

A second perplexing observation between the Israeli cities can be seen from examining the estimates of *R*_e_ for two very different cities such as Bnei Brak and Tel Aviv. As opposed to Tel-Aviv Bnei Brak has large ultra-orthodox religious communities that are characterized by large families with many children. The two cities differ in many important demographic aspects (e.g. household size, age structure and population connectivity) that are thought to influence the dynamics of influenza [47]–[49]. It is expected that significant variation in demographic factors would lead to observed differences in the value of a key parameter such as ***R*** **_e_**. During our study period there were no statistical differences between the value of *R_e_* between different cities (within a year), and even countries. The fact that the size of ***R*** **_e_** is rather “constant” between the Israeli cities is in line with the findings of Baroyan et al (1977) [50], Spicer (1979) [20] and Chowell (2010) [21] which were obtained on the much larger geographical scale of the USSR and Brazil. Spicer (1979) remarks: “The most striking and unexpected feature of the model is that the parameter on which the spread of an epidemic within a city depends is the same for every city in any one epidemic within the USSR” [20]. Nevertheless, in recent years extremely complicated models were developed to model pandemic influenza [47]–[49], [51]. The models include many biological demographic and sociological complexities which are believed to be important in capturing the dynamics of pandemic influenza. For instance, Merler et al 2011 [51] had to incorporate information on intra-European mobility and the different socio-demographic structure of the different European countries in order to reproduce the observed spatial pattern of the West to East spread of the 2009 pandemic in Europe. In contrast, demographic structure did not appear to have impact on the spread of influenza in Israel.

Additional data on the spatial spread of influenza combined with statistical analysis is required to better understand how different population demographics effect *R_e_* and the propagation of the disease within different communities.

## Methods

### Data

Our dataset consisted of all Influenza-Like Illness (ILI) cases in Israel diagnosed daily by Maccabi Health Services doctors, between January 1^st^ 1998 and May 31^st^ 2009. Diagnosis codes included in this database are ICD9 code 487.1 (influenza) and internal Maccabi codes for influenza, influenza-like disease and swine influenza. The last year of data, in which the swine flu pandemic occurred, was excluded. ICD9 codes were used exclusively in 1998–2002, when there was a transition to internal diagnosis codes. The ILI data were corrected for repeat visits. The criterion chosen to filter out repeat visits from the data was recommended by the Israeli Center for Disease Control, and defines a visit as a repeat visit if it comes within 28 days of a pervious visit with ILI symptoms. The data exhibits a strong weekly cycle due to the absence of weekend reporting. Hence, the data was smoothed using a 7-day moving average kernel [52] red line in Figure 1A. The dataset includes 7 seasons in which the dominant strain was influenza A H3N2, two seasons in which the dominant strain was influenza B, one season in which the dominant strain was influenza A H1N1, and one season in which no strain was dominant. Historical data of dominant influenza strains in Israel is available at the World Health Organization's FluNet website at http://apps.who.int/globalatlas/dataQuery/default.asp.Maccabi is the second-largest Health Maintenance Organization (HMO) in Israel and insures about 23% of Israel's population. During the period analyzed the number of Maccabi members varied between 1.37 and 1.86 million people. Several works based on this dataset have already been published [30], [53], [54]. The French ILI dataset is taken from the Sentinel network - a network of ∼1200 General Practitioners (GPs) in France who, since 1984, regularly collect data about diagnoses of 12 diseases and report it via the internet. ILI is defined in the Sentinel network as sudden temperatures greater than 39°C, myalgia and cough/running nose. The data received from the GPs are then processed to estimate the number of ILI cases per 100,000 residents in each region, by using population data and the fraction of GPs taking part in the surveillance out of the total number of GPs. Since the frequency of reporting is irregular and is left for each doctor to decide, the data presented in the Sentinel network website are weekly aggregate incidences [55].

### Model

We used an SIR discrete-time age-of-infection model as described in [56]. The total population is denoted by *N*. The number of susceptibles at the end of day *t* is denoted by *S(t)* while the number of people who become infected on day *t* is denoted by *i(t)*. It is important to emphasize that *i(t)* here counts only the newly-infected individuals on day *t*. Note the key relationship:
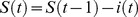
(4)


It is assumed that each individual has, on average, *β* contacts with other random individuals per day. A person who becomes infective retains a certain (and non-constant) degree of infectivity for *d* days. The number of days since a person's infection is termed its age of infection. Therefore, the number of infectives whose age of infection is *τ (1≤τ≤d)* on day *t* is *i(t−τ)*. When a susceptible meets an infective person whose age-of-infection is *τ (1≤τ≤d)*, the susceptible becomes infective with probability *P_τ_*. The vector *P = (P_1_; …; P_d_)* thus defines the infectivity profile, and is a key parameter of the model [16]. The values for the vector *P* were obtained from the comprehensive review paper about influenza viral shedding by Carrat et al 2008 [57]. The values are *P* = (0.073, 0.181, 0.222, 0.185, 0.137, 0.09, 0.056, 0.032, 0.016, 0.008).

The probability that any single susceptible becomes infected during day *t* is given by:
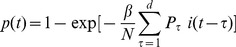
(5)In a deterministic variation of the model, the daily number of infectives is, for large *N*:
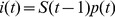
(6)We use the deterministic model to simulate the time-series of figure 3.

The above model also has a stochastic formulation (Katriel et al. 2011) whose log-likelihood can be shown to be (Katriel, manuscript):
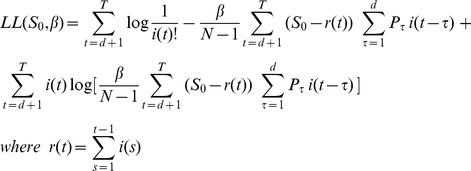
(7)It is possible to estimate the parameters *S_0_* and *R_0_* by numerically maximizing the above log-likelihood expression. The maximization should be carried out for β>0 and over integers *S_0_* in the range 
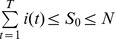
 (as the number of susceptibles at the beginning cannot be smaller than the total number of cases).

**Figure 5:**
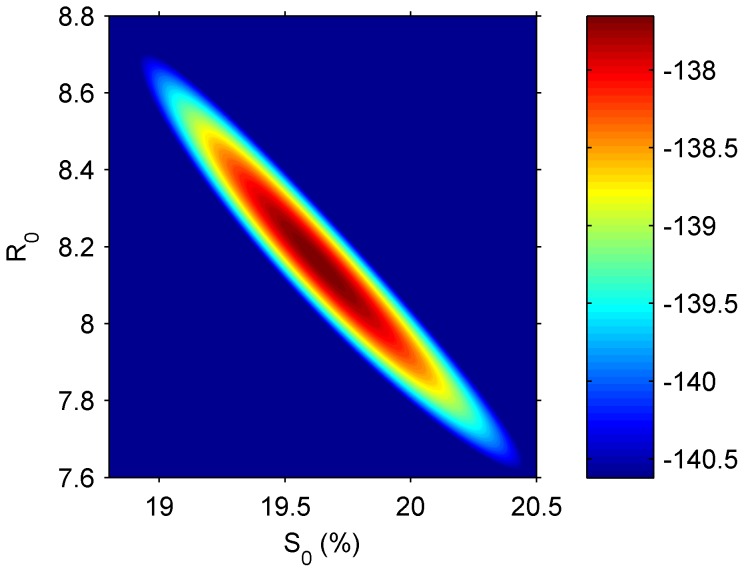
A map of likelihood values as a function of *S*_0_ and *R*_0_ in the 2006–2007 season. The colored region includes the sets of parameters giving the maximum likelihood and likelihoods which are up to 3 units below the maximum likelihood. The upper and lower limits of this region were used as a 95% confidence interval for the *S*_0_ and *R*_0_ values [58].

It is important to demonstrate the statistical identifiability of the parameters *S_0_* and *R_0_* from the data, using the likelihood function 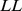 (equation 7) [58].To achieve this, we generated contour plots of the function 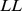 for each season. Figure 5 displays this plot for season 9. As can be observed, the likelihood attains a unique maximum at a point which forms our maximum likelihood estimates, and the region in which the likelihood is close to the maximal values is small enough to provide rather narrow 95% confidence intervals for the parameters. The corresponding plots for other seasons are qualitatively similar.

In another approach that was taken to obtain bootstrap confidence intervals for the effective reproduction number (*R*_e_), the stochastic version of the model (equations 4–6) was simulated 10,000 times using the exact same parameters (i.e., *β* and *S*_0_ where estimated from the real data using (equation 7)). For each of the simulated epidemics *R_e_* was re-estimated and the 250 lowest and highest estimates were removed to give the 95% bootstrap confidence intervals.

The values of *R_e_* were also obtained using the classic method of measuring the rate of exponential growth at the initiation of the outbreak as in [32] The estimates from both methods were highly correlation (R = 0.91, p = 0.0007) (see table 1).
